# Integrated downstream regulation by the quorum-sensing controlled transcription factors LrhA and RcsA impacts phenotypic outputs associated with virulence in the phytopathogen *Pantoea stewartii* subsp. *stewartii*

**DOI:** 10.7717/peerj.4145

**Published:** 2017-12-06

**Authors:** Duy An Duong, Ann M. Stevens

**Affiliations:** Department of Biological Sciences, Virginia Polytechnic Institute and State University (Virginia Tech), Blacksburg, VA, United States of America

**Keywords:** LrhA, *Pantoea stewartii* subsp. *stewartii*, Phytopathogen, Quorum sensing, RcsA, Transcription factor

## Abstract

*Pantoea stewartii* subsp. *stewartii* is a Gram-negative proteobacterium that causes leaf blight and Stewart’s wilt disease in corn. Quorum sensing (QS) controls bacterial exopolysaccharide production that blocks water transport in the plant xylem at high bacterial densities during the later stage of the infection, resulting in wilt. At low cell density the key master QS regulator in *P. stewartii*, EsaR, directly represses *rcsA*, encoding an activator of capsule biosynthesis genes, but activates *lrhA*, encoding a transcription factor that regulates surface motility. Both RcsA and LrhA have been shown to play a role in plant virulence. In this study, additional information about the downstream targets of LrhA and its interaction with RcsA was determined. A transcriptional fusion assay revealed autorepression of LrhA in *P. stewartii* and electrophoretic mobility shift assays (EMSA) using purified LrhA confirmed that LrhA binds to its own promoter. In addition, LrhA binds to the promoter for the RcsA gene, as well as those for putative fimbrial subunits and biosurfactant production enzymes in *P. stewartii,* but not to the *flhDC* promoter, which is the main direct target of LrhA in * Escherichia coli.* This work led to a reexamination of the physiological function of RcsA in *P. stewartii* and the discovery that it also plays a role in surface motility. These findings are broadening our understanding of the coordinated regulatory cascades utilized in the phytopathogen *P. stewartii*.

## Introduction

*Pantoea stewartii* subsp. *stewartii*, a Gram-negative rod-shaped, gamma-proteobacterium, is the causal agent of leaf blight and Stewart’s wilt in susceptible varieties of *Zea mays*. It is primarily transmitted to the plant by the corn flea beetle, *Chaetocnema pulicaria* (Esker & Nutter, 2002). After being deposited through excrement into wounds generated during insect feeding, the pathogen gains access to the leaf apoplast and causes water-soaked lesions through the Hrp-type III secretion system (Ham et al., 2006; Roper, 2011). In a second phase of the disease, the bacteria then also migrate to the xylem, where they grow to high cell density and form a biofilm that blocks water flow within the plant. This results in wilt disease and even death, if the plants were infected at the seedling phase (Braun, 1982). Quorum sensing (QS), a mechanism of bacterial cell density-dependent communication, controls the virulence, capsule production and surface motility of this pathogen (Roper, 2011; Von Bodman, Bauer & Coplin, 2003).

During QS, *P. stewartii* produces N-acyl homoserine lactone (AHL) signals due to the activity of EsaI, a LuxI-type protein (Beck von Bodman & Farrand, 1995). The AHL signal then interacts with the master QS regulatory protein EsaR, a LuxR homologue, when the cell density reaches a critical threshold. EsaR is a dual-level transcriptional regulator that binds to DNA at its recognition sites to either activate or repress its downstream targets at low cell density (Beck von Bodman & Farrand, 1995; Von Bodman et al., 2003; Von Bodman, Majerczak & Coplin, 1998). When EsaR and AHL interact at high cell density, the EsaR-AHL complex is unable to bind to the DNA resulting in transcriptional deactivation or derepression of its target genes (Schu et al., 2009; Shong et al., 2013). Multiple approaches have been used to identify several direct targets of EsaR, including classic genetic (Minogue et al., 2005), proteome-level (Ramachandran & Stevens, 2013) and transcriptome-level (Ramachandran et al., 2014) analysis. Two of these direct targets, *rcsA* and *lrhA*, are involved in plant virulence and control capsule production and surface motility, respectively (Kernell Burke et al., 2015).

EsaR directly represses the *P. stewartii rcsA* gene at low cell density, insuring precise control over the timing of capsule synthesis (Carlier & Von Bodman, 2006; Minogue et al., 2005; Von Bodman, Majerczak & Coplin, 1998). At high cell density, gene activation by RcsA leads to production of stewartan, a polymer of galactose, glucose and glucuronic acid in a 3:3:1 ratio, which is the main component of the exopolysaccharide (EPS) (Nimtz et al., 1996). Stewartan is a primary virulence factor of *P. stewartii* (Carlier, Burbank & Von Bodman, 2009; Minogue et al., 2005; Roper, 2011). Previous work has shown that the *lrhA* gene is directly activated by EsaR at low cell density and a *P. stewartii* LrhA deletion mutant exhibits decreased surface motility and intermediate virulence levels in comparison to the wild type (Kernell Burke et al., 2015). However, little is known about the precise role of LrhA and its targets with regard to surface motility and virulence in *P. stewartii*.

In *Escherichia coli*, the function of LrhA is better understood. It is the key regulator controlling the expression of flagella, motility and chemotaxis by regulating the synthesis of FlhD_2_C_2_, the master regulator of flagella and chemotaxis gene expression (Lehnen et al., 2002). In *E. coli*, LrhA directly activates its own expression and represses the expression of *flhD/flhC*, thereby suppressing motility and chemotaxis (Lehnen et al., 2002). LrhA also controls *fimA* expression (Blumer et al., 2005) and regulates RpoS translation (Gibson & Silhavy, 1999; Peterson et al., 2006), but its binding site is not well defined (Lehnen et al., 2002).

In contrast to *E. coli*, *P. stewartii* is only capable of swarming rather than swimming motility. The bacterium’s swarming motility is controlled by QS and contributes to its pathogenicity (Herrera et al., 2008). The surface motility is flagellar-dependent since deletion of *fliC*_*I*_ renders the bacterium aflagellar and incapable of swarming (Herrera et al., 2008). There is no evidence demonstrating that EsaR plays a direct role in regulating flagella synthesis. However, EsaR does directly regulate *lrhA* in *P. stewartii* and thereby indirectly regulates surface motility and plant virulence (Kernell Burke et al., 2015) through unknown mechanisms. A transcriptome-level analysis of the LrhA regulon in *P. stewartii* showed that LrhA activates three genes and represses 23 genes four-fold or more (Kernell Burke et al., 2015). In the present study, *CKS_0458* and *CKS_5211*, genes putatively encoding a fimbrial subunit and biosurfactant production enzyme, respectively, have now been confirmed to be direct targets of LrhA. In addition, LrhA has also been demonstrated to repress its own gene and that of RcsA. Follow-up studies led to the finding that RcsA also plays a role in surface motility. This work has helped further reveal how the QS regulatory cascade in *P. stewartii* coordinately controls genes important for interactions with the plant host*.*

## Materials and Methods

### Strains and growth conditions

Table 1 lists strains and plasmids used in this study. *E. coli* strains were grown in Luria-Bertani (LB) (10 g/l tryptone, 5 g/l yeast extract, and 5 g/l NaCl) broth or plates with 1.5% agar at 37 °C. *P. stewartii* strains were grown in either LB or Rich Minimal (RM) medium (1 × M9 salts, 2% casamino acids, 1 mM MgCl_2_, and 0.4% glucose) at 30 °C. Growth medium was supplemented with the following antibiotics: ampicillin (Ap, 100 µg/ml), chloramphenicol (Cm, 35 µg/ml), kanamycin (Kn, 50 µg/ml), nalidixic acid (Nal, 30 µg/ml), or streptomycin (Str, 100 µg/ml) as required (see Table 1).

### Green fluorescent protein fusion (GFP) construction and testing

A transcriptional fusion between the *lrhA* promoter (903 bp) and the gene for GFP was created through traditional molecular techniques as described previously (Kernell Burke et al., 2015). PCR primers (Table S1) with the restriction sites *Eco*RI and *Kpn*I added to the 5′ and 3′ ends of the promoter sequence, respectively, were used to facilitate subcloning into the pPROBE′-GFP [tagless] vector (Miller, Leveau & Lindow, 2000). *E. coli* S17-1 was transformed with this plasmid construct containing P_*lrhA*_-*gfp*, which was then moved into the wild-type *P. stewartii* DC283, Δ*lrhA* and Δ*lrhA/lrhA*^+^ strains (Table 1) via conjugation. The transconjugates were grown in RM medium supplemented with Kn and Nal overnight, diluted in fresh medium to an OD_600_ of 0.05 at 30 °C with shaking at 250 rpm to an OD_600_ of 0.2–0.5, diluted a second time in fresh medium to an OD_600_ of 0.025 and grown to an OD_600_ of 0.5. GFP measurements were done as previously described (Kernell Burke et al., 2015) with average relative fluorescence/OD_600_ from three experiments of triplicate samples, standard errors, and two-tailed homoscedastic Student’s *t*-test values calculated for each strain.

**Table 1:** Strains and plasmids used in the study.

**Strains**	**Genotype and notes**^a^	**References**
***Pantoea stewartii*strains**		
DC283	Wild-type strain; Nal^*r*^	Dolph, Majerczak & Coplin (1988)
Δ*lrhA*	Unmarked deletion of *lrhA* coding sequence from DC283; Nal^*r*^	Kernell Burke et al. (2015)
Δ*lrhA/lrhA*^+^	Δ*lrhA* with chromosomal complementation of *lrhA* and its promoter downstream of *glmS*; Nal^*r*^ Cm^*r*^	Kernell Burke et al. (2015)
Δ*rcsA*-2015	Unmarked deletion of *rcsA* coding sequence from DC283; Nal^*r*^, missing 66-kb region	Kernell Burke et al. (2015)
Δ*rcsA/rcsA*^+^-2015	Δ*rcsA* with chromosomal complementation of *rcsA* and its promoter downstream of *glmS*; Nal^*r*^ Cm^*r*^, missing 66-kb region	Kernell Burke et al. (2015)
Δ*rcsA*-2017	Unmarked deletion of *rcsA* coding sequence from DC283; Nal^*r*^	This study
Δ*rcsA/rcsA*^+^-2017	Δ*rcsA* with chromosomal complementation of *rcsA* and its promoter downstream of *glmS*; Nal^*r*^ Cm^*r*^	This study
Δ*CKS_0458-CKS_0459*	Unmarked deletion of both *CKS_0458* and *CKS_0459* coding sequence from DC283; Nal^*r*^	This study
Δ*CKS_0458-CKS_0459 /CKS_0458*^+^	Δ*CKS_0458-CKS_0459* with chromosomal complementation of *CKS_0458* and its promoter downstream of *glmS*; Nal^*r*^ Cm^*r*^	This study
Δ*CKS_0458-CKS_0459 /CKS_0458-CKS_0459*^+^	Δ*CKS_0458-CKS_0459* with chromosomal complementation of *CKS_0458-CKS_0459* and their promoter downstream of *glmS*; Nal^*r*^ Cm^*r*^	This study
Δ*CKS_5208*	Unmarked deletion of *CKS_5208* coding sequence from DC283; Nal^*r*^	This study
Δ*CKS_5208/CKS_5208*^+^	Δ*CKS_5208* with chromosomal complementation of *CKS_5208* and its promoter downstream of *glmS*; Nal^*r*^ Cm^*r*^	This study
Δ*CKS_5211*	Unmarked deletion of *CKS_5211* coding sequence from DC283; Nal^*r*^	This study
Δ*CKS_5211/CKS_5211*^+^	Δ*CKS_5211* with chromosomal complementation of *CKS_5211* and its promoter downstream of *glmS*; Nal^*r*^ Cm^*r*^	This study
Δ*CKS_5211/*Δ*CKS_5208*	Unmarked deletion of both *CKS_5211* and *CKS_5208* coding sequences from DC283; Nal^*r*^	This study
***Escherichia coli*strains**		
Top 10	*F*^−^*mcrA*Δ(*mrr-hsdRMS-mcrBC*) Φ*80dlacZ*Δ*M15*Δ*lacX* 74 *deoR recAI araD139*Δ(*ara-leu)7697 galU galK rpsL (* Str^*r*^*) endA1 nupG*	Grant et al. (1990)
DH5 *αλpir*	*F*^−^*endA1 glnV44 thi-1 recA1 relA1 gyrA96 deoR nupG*Φ*80dlacZ*Δ*M15*Δ*(lacZYA-argF)U169 hsdR17(rK- mK+)λpir*	Kvitko et al. (2012)
S17-1	* recA pro hsdR RP4-2-Tc::Mu-Km::Tn7*	Simon, Priefer & Pühler (1983)
S17-1 *λpir*	*recA pro hsdR RP4-2-Tc::Mu-Km::Tn7λpir*	Labes, Puhler & Simon (1990)
BL21-DE3	*fhuA2 [lon] ompT gal* (*λ DE3*) *[dcm]*Δ*hsdSλ DE3*=*λ sBamHIo*Δ*EcoRI-B int::(lacI::PlacUV5::T7 gene1) i21*Δ*nin5*	Studier & Moffatt (1986)
**Plasmids**		
pGEM-T	Cloning vector, Ap^*r*^	Promega
pET28a	Expression vector, Kn^*r*^	Novagen
pDONR201	Entry vector in the Gateway system, Kn^*r*^	Life Technologies
pAUC40	Suicide vector pKNG101::*attR*-*ccdB*-Cm^R^; Cm^*r*^, Str^*r*^, *sacB*	Carlier, Burbank & Von Bodman (2009)
pEVS104	Conjugative helper plasmid, *tra trb;* Kn^*r*^	Stabb & Ruby (2002)
pUC18R6K-mini-Tn7-cat	Tn7 vector for chromosomal integration into the intergenic region downstream of *glmS*; Cm^*r*^, Ap^*r*^	Choi et al. (2005)
pPROBE′-GFP[tagless] P_*lrhA*_	pPROBE′-GFP[tagless] vector with the promoter of *lrhA;* Kn^*r*^	This study

**Notes.**

a
Ap^*r*^ ampicillin resistance Nal^*r*^ nalidixic acid resistance Kn^*r*^ kanamacyin resistance Cm^*r*^ chloramphenicol resistance Str^*r*^ streptomycin resistance

### Overexpression of LrhA

The *lrhA* coding sequence was amplified using primers with *Bam*HI and *Hin*dIII sites (Table S1), cloned into pGEM-T (Promega, Madison, WI, USA), and sequenced. After double digestion with *Bam*HI and *Hin*dIII, the construct was ligated into pET28a (Novagen, Madison, WI, USA) and transformed into *E. coli* (BL21-DE3) (Studier & Moffatt, 1986) to express LrhA with a His_6_ tag at the N-terminus (37 kDa). Induction of protein expression with 0.1 M isopropyl *β*-D-1-thiogalactopyranoside (IPTG) was performed at an OD_600_ of 0.5–0.8, 19 °C, overnight, shaking at 250 rpm. Cells were pelleted by centrifugation at 5,000 rpm in a JA-10 rotor (Beckman Coulter, Brea, CA, USA) for 20 min at 4 °C, snap-frozen with liquid nitrogen and stored at −75 °C. The cell pellet was then resuspended in Ni-NTA wash buffer (50 mM Tris–HCl, 300 mM NaCl, 50 mM imidazole) and sonicated to release proteins. Ultracentrifugation at 40,000 rpm in a Beckman Ti70 rotor for 1 h at 4 °C was used to subsequently remove the cell debris. The protein was purified using a Ni-NTA column (HisTrap HP, GE Healthcare) with Ni-NTA elution buffer (50 mM Tris–HCl, 300 mM NaCl, 500 mM imidazole). The protein purity was observed through standard SDS-PAGE electrophoresis.

### Electrophoretic mobility shift assays (EMSA)

Promoter regions of genes of interest were amplified with FAM-labeled primers (Table S1) and extracted from a 1% agarose gel to examine the specific binding with purified His_6_-LrhA over a range of concentrations. Twenty µl reactions with purified His_6_-LrhA, 5 nM FAM-DNA in 1X EMSA buffer (10% glycerol, 1 mM MgCl_2_, 0.5 mM EDTA, 0.5 mM DTT, 50 mM NaCl, 10 mM Tris–HCl, 50 µg/ml poly (dI-dC) and 150 µg/ml BSA) were incubated at room temperature for 1 h before loading on to 1 × TBE (10.8 g/l Tris–HCl, 5.5 g/l boric acid, 2 mM EDTA, pH 8.0) 4%, 5%, or 6% acrylamide native gels followed by electrophoresis at 80 V for 2–3 h. Images were visualized on a Typhoon Trio Scanner (GE Healthcare). Experiments were done in duplicate.

### Construction of unmarked deletion mutant strains

Chromosomal deletions of *CKS_0458/CKS_0459, CKS_5208*, *CKS_5211*, and *CKS_5211/CKS_5208* were constructed based on the Gateway system (Life Technologies) and suicide vectors as described previously (Kernell Burke et al., 2015), but with primers listed in Table S1. In addition, another chromosomal deletion of *rcsA* was constructed using the same approach as described in a previous study (Kernell Burke et al., 2015), due to a deletion of ∼66-kilobases (kb) in the chromosome of the original construct.

### Construction of chromosomal complementation strains

Complementation strains were constructed by generating a chromosomal insertion of the promoter and coding regions of the target gene into the neutral region downstream of *glmS* on the *P. stewartii* chromosome using the pUC18R6K-mini-Tn7-cat vector system (Choi et al., 2005) as previously described (Kernell Burke et al., 2015), but with primers listed in Table S1.

### Phenotypic surface motility assay

Swarming motility for the wild-type, deletion and complementation strains was investigated under strict conditions to ensure a reproducible phenotype as previously described (Kernell Burke et al., 2015). Briefly, five µl of cell culture at an OD_600_ of 0.5 were spotted directly on the agar surface of LB 0.4% agar quadrant plates supplemented with 0.4% glucose (Herrera et al., 2008). Plates were incubated in a closed plastic box at 30 °C for 2 days prior to observation.

### Phenotypic capsule production assay

Bacterial strains were grown overnight in LB broth supplemented with the appropriate antibiotics at 30 °C with shaking. The overnight cultures were subcultured in fresh LB medium to an OD_600_ of 0.05 and grown to an OD_600_ of 0.5 at 30 °C with shaking. The strains were then cross-streaked with sterilized wooden sticks on 1.5% agar plates containing 0.1% casamino acids, 1% peptone and 1% glucose (CPG) (Kernell Burke et al., 2015; Von Bodman, Majerczak & Coplin, 1998). Plates were incubated at 30 °C, lid-up for 2 days to observe the capsule production and visualized using the Bio-Rad Gel Doc imager system.

### Plant virulence assay

Virulence assays with *P. stewartii* strains in *Zea mays* seedlings were adapted from established methods (Von Bodman, Majerczak & Coplin, 1998; Kernell Burke et al., 2015) with some modifications. In this study, *Zea mays* cv. Jubilee, 2B seeds were planted in Sunshine mix #1 or Promix soil for seven or six days, respectively, in a 28 °C growth chamber with ∼100–200 µE m^−2^ s^−1^ light intensity, 16 h light/8 h dark and ∼80% relative humidity (Percival Scientific, Inc.). Fifteen seedlings between 6 and 10 cm of height with two separated leaves were inoculated with five µl (∼3 × 10^5^ CFU) of bacterial culture grown to an OD_600_ of 0.2 in LB broth (∼6 × 10^7^ CFU/ml). Prior to plant inoculation, the bacterial cells were washed and resuspended in phosphate buffered saline (PBS; 137 mM NaCl, 2.7 mM KCl, 10 mM Na_2_HPO_4_ and 2 mM KH_2_PO_4_, pH 7.4). Wild-type strain and PBS controls were included in each trial, then, accumulated numbers of control-inoculated plants across all experiments were analyzed. A sterile needle (26G 5/8, 15.9 mm, SUB-Q Becton, Dickinson and Company) was used to make an ∼1 cm incision in the stem ∼1 cm above the soil line and the bacteria were added to the plant by slowly pipetting the inoculum while moving across the wound five times. The plants were observed on day 12 post-infection to assess the virulence by two independent observers. Symptom severity was scored based on a five-point scale with 0 = no symptoms; 1 = few scattered lesions; 2 = scattered water soaking symptoms; 3 = numerous lesions and slight wilting; 4 = moderately severe wilt; 5 = death. The data for each treatment were averaged together and used to calculate the mean and standard error. A Student’s *t*-test was used to calculate the *p*-value for experimental treatments compared to the wild-type treatment.

## Results

### LrhA autorepresses its own gene expression in *P. stewartii*

**Figure 1:**
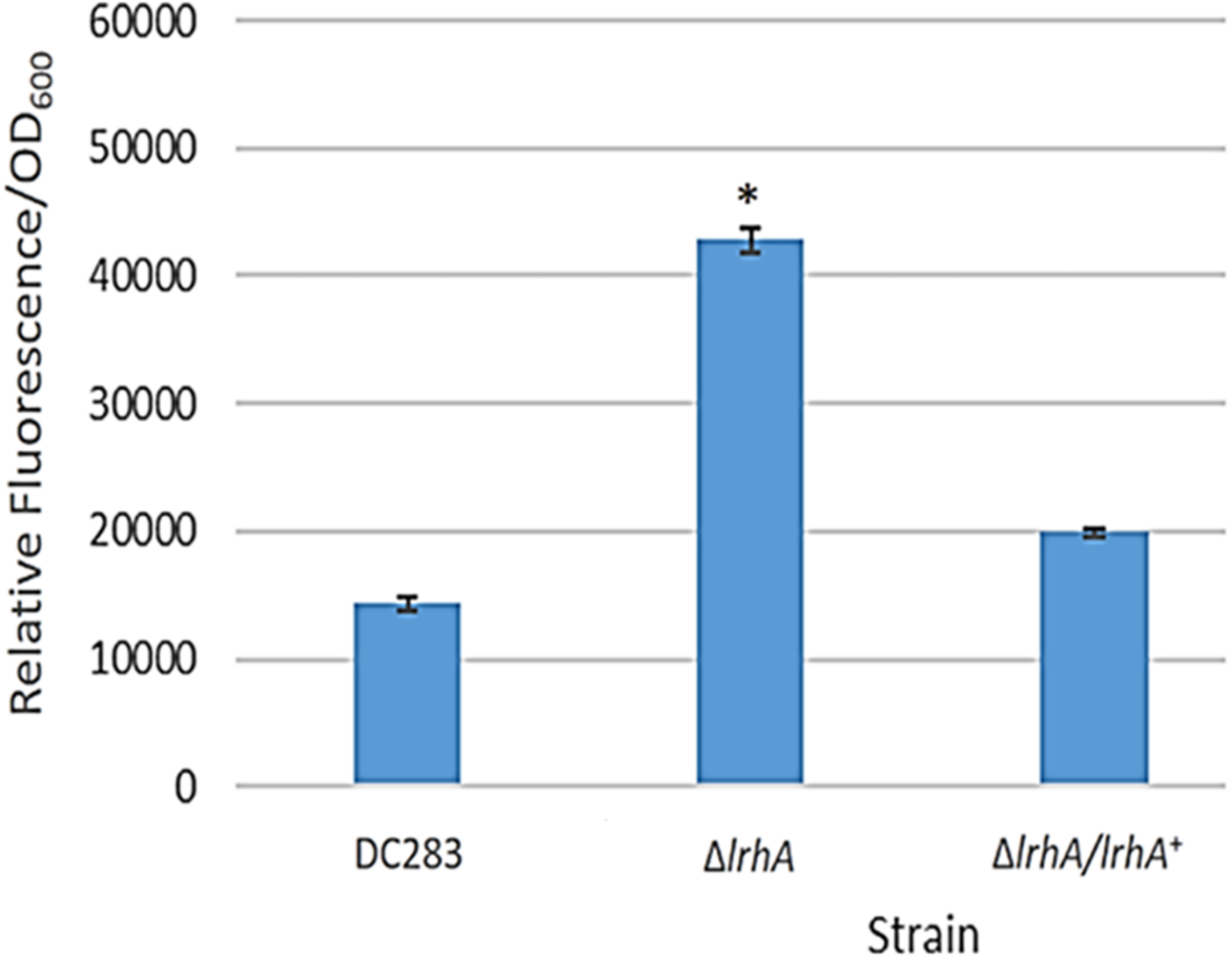
Expression levels of a *lrhA* promoter-*gfp* transcription reporter in three *P. stewartii* strains. The wild-type DC283 and Δ*lrhA* and Δ*lrhA/lrhA*^+^ strains in the same genetic background (containing pPROBE′-GFP[tagless] P_*lrhA*_) were grown to an OD_600_ of 0.5 and GFP expression levels from the *lrhA* promoter-*gfp* transcription reporter were measured as average relative fluorescence/OD_600_. Data represents three experimental samples analyzed in triplicate. Error bars denote standard error. The asterisk (∗) represents a statistically significant difference (*p* < 0.05) between the Δ*lrhA* and both the wild-type and Δ*lrhA/lrhA*^+^ strains using a two-tailed homoscedastic Student’s *t*-test.

A GFP reporter was used to measure levels of transcription from the *lrhA* promoter in the wild-type, Δ*lrhA* and Δ*lrhA/lrhA*^+^ strains of *P. stewartii* DC283 (Table 1). Expression levels of GFP in the Δ*lrhA* strain were significantly higher than the wild-type strain (Fig. 1, *p* = 0.00001) indicating that LrhA normally represses its own expression in the wild-type strain. The expression level of the *lrhA* promoter in the complementation Δ*lrhA/lrhA*^+^ strain was restored to levels closer to those of the wild-type strain, and was also significantly different than the deletion strain (Fig. 1, *p* = 0.00002).

**Figure 2:**
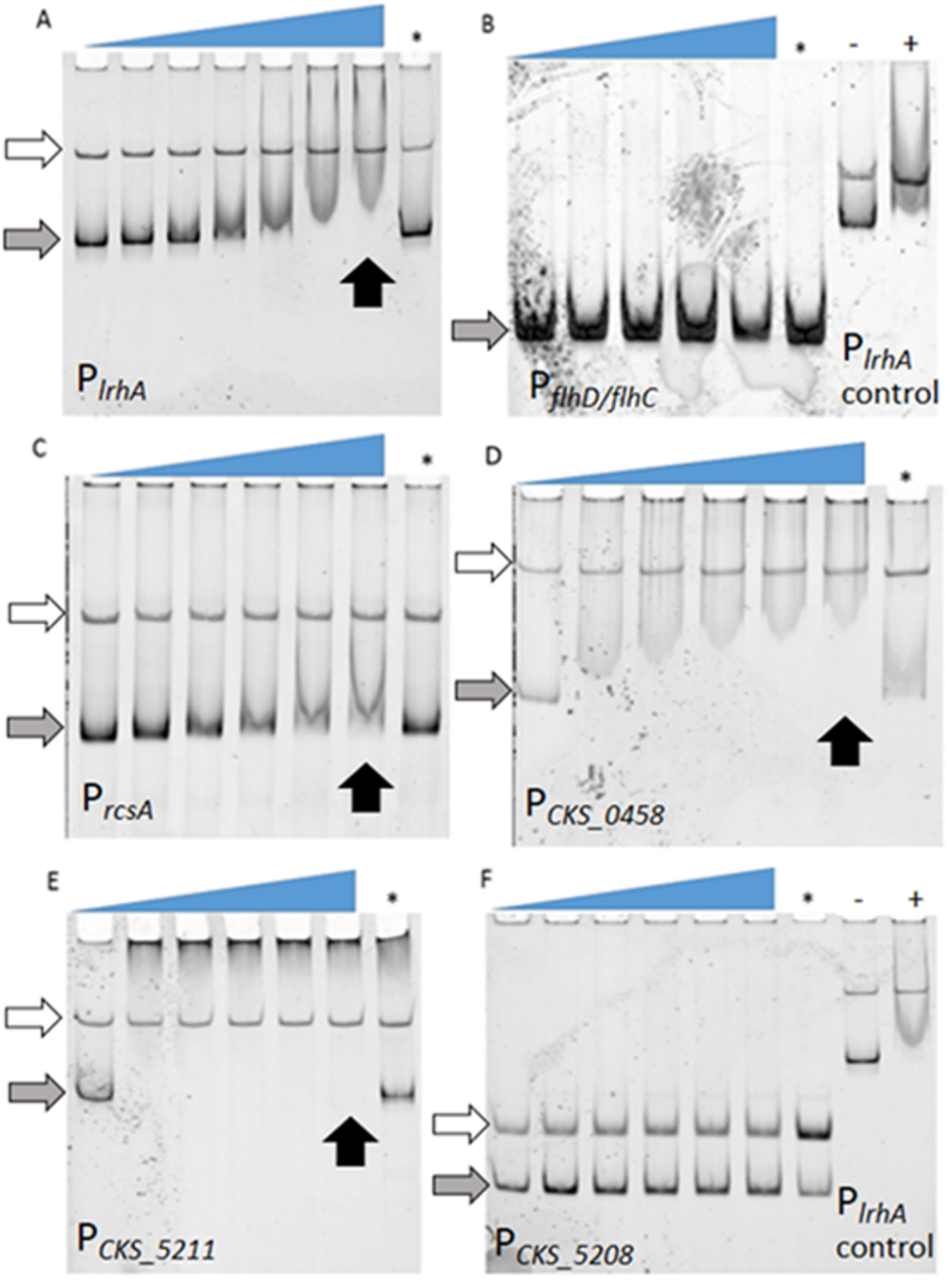
Examination of binding of LrhA to select target promoters via EMSA. FAM-DNA probes were incubated with increasing concentrations of His_6_-LrhA (LrhA) from left to right, corresponding to the slope of the triangles, to investigate the mobility shift upon specific binding to the protein. The competition reaction (indicated by the asterisk, ∗) was conducted with 25 nM unlabeled DNA of P_*lrhA*_ to prove the specificity of the interaction. Autoregulation of LrhA was confirmed with the direct binding between purified LrhA to its promoter (A). Shifted bands were also observed with P_*rcsA*_ (C), P_*CKS_0458*_ (D), and P_*CKS_5211*_ (E). There were no shifted bands observed for P_*flhDC*_ (B) and P_*CKS_5208*_ (F), while the positive controls for LrhA activity showed a shift (−: reaction with P_*lrhA*_ probe in the absence of LrhA, +: reaction with P_*lrhA*_ probe in the presence of 200 nM LrhA). Concentrations of LrhA tested for P_*lrhA*_ (A) are 0, 25, 50, 100, 200, 400, and 800 nM. Concentrations of LrhA tested for P_*flhDC*_ (B) are 0, 400, 600, 800, and 1,000 nM. Concentrations of LrhA tested for P_*rcsA*_ (C), P_*CKS_0458*_ (D), P_*CKS_5211*_ (E) and P_*CKS_5208*_ (F) are 0, 200, 400, 600, 800, and 1,000 nM. Grey arrows highlight unbound DNA probes. White arrows indicate unbound DNA generated during PCR reactions that do not interact specifically with LrhA. Black arrows point to the lane with specific binding at the highest concentration of LrhA.

### Identification of LrhA direct targets through EMSAs

To determine if the observed *lrhA* autorepression occurred directly or indirectly, electrophoretic mobility shift assays (EMSAs) were performed. First, direct binding of LrhA to the promoter of its own gene was demonstrated by EMSA analysis (Fig. 2A). Next, the ability of LrhA to directly regulate additional gene targets was explored, using the *lrhA* promoter as a positive control for the His_6_-LrhA activity and unlabeled P_*lrhA*_ DNA to prove the specificity of the binding. In *E. coli*, LrhA is known as a repressor of motility by direct interaction with the promoter region of *flhD/flhC*, whose products promote the expression of flagellar gene synthesis (Lehnen et al., 2002). However, RNA-Seq data of expression levels of *flhD/flhC* in *P. stewartii* showed less than a two-fold difference between wild-type and Δ*lrhA* strains (Kernell Burke et al., 2015) suggesting a lack of transcriptional regulation. Here, EMSA analysis showed that His_6_-LrhA does not bind to the promoter of *flhD/flhC* (Fig. 2B), explaining the observed lack of transcriptional regulation*.*

Additional analysis of the LrhA-regulated transcriptome in *P. stewartii* revealed that LrhA repressed the expression level of several more downstream targets, including *rcsA, CKS_0458*, *CKS_5208* and *CKS_5211* (Kernell Burke et al., 2015). RcsA activates capsule production, a known virulence factor in *P. stewartii* (Kernell Burke et al., 2015; Minogue et al., 2005; Poetter & Coplin, 1991; Wehland et al., 1999). The putative roles of genes for fimbria encoded by *CKS_0458*, annotated as a putative fimbrial subunit, (and *CKS_0459* located downstream in an operon) and for surfactant expression encoded by *CKS_5208* and *CKS_5211*, annotated as a rhamnosyltransferase I subunit B and a putative alpha/beta superfamily hydrolase/acyltransferase, respectively, in plant colonization and/or virulence had not been established. However, it seemed plausible that they might also play roles in host association as they were some of the most highly LrhA-repressed genes, four-fold or greater (Kernell Burke et al., 2015). Therefore, the binding of LrhA to the promoters of these genes was also examined via EMSA. The direct binding of His_6_-LrhA to P_*rcsA*_*,* P_*CKS*_0458_ and P_*CKS*_5211_ (Figs. 2C–2E), was demonstrated via EMSAs while P_*CKS*_5208_ did not interact with His_6_-LrhA *in vitro* (Fig. 2F). Collectively, these findings identified four directly controlled gene targets in the LrhA regulon. The lack of LrhA regulation of FlhD_2_C_2_, the master regulator of flagellar-based motility and chemotaxis in *E. coli*, indicates a different role for LrhA in controlling *P. stewartii* motility. The direct binding of LrhA to the promoter of *rcsA* further links LrhA to *P. stewartii* pathogenesis. The role of the two other LrhA direct targets *CKS_0458* and *CKS_5211* remained to be established.

### Examining the role of putative fimbrial and surfactant production genes in the surface motility and virulence of *P. stewartii*

**Figure 3:**
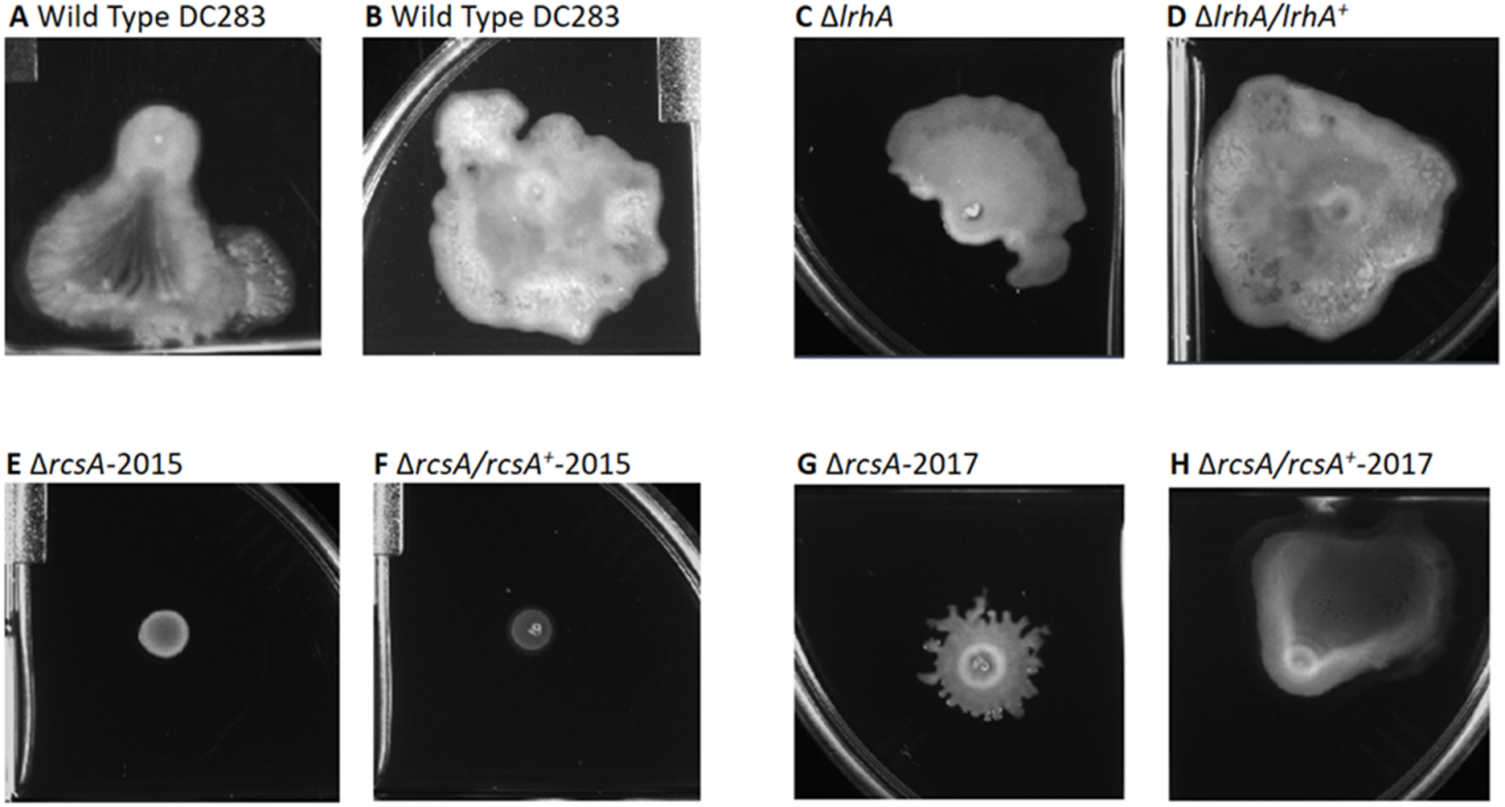
Impact of RcsA and LrhA on surface motility of *P. stewartii*. The pictures show the analysis of surface motility in *P. stewartii* DC283 strains. Examples of wild type unidirectional (A) or omnidirectional surface motility (B) are shown as controls. The Δ*lrhA/lrhA*^+^ complementation strain (D) is similar to the control in (B), while the Δ*lrhA* strain has reduced surface motility expanding over a smaller surface area (C), as has been previously observed (Kernell Burke et al., 2015). Both Δ*rcsA* strains had dramatically reduced surface motility (E and G) as well as the Δ*rcsA/rcsA*^+^-2015 strain (F). The Δ*rcsA/rcsA*^+^-2017 strain was complemented for the defect in surface motility (H). All pictures were taken at the same magnification after 2 days of incubation at 30 °C in a closed plastic box.

To further investigate the role of the downstream targets of LrhA putatively involved in production of fimbriae and surfactant, a reverse genetic approach was utilized. Markerless deletions of *CKS_0458/CKS_0459, CKS_5208*, *CKS_5211* and *CKS_5211*/*CKS_5208* were successfully generated. Corresponding chromosomal complementation strains were also generated with the exception of a double deletion mutant of *CKS_5211*/*CKS_5208* complementation strain, due to the length constraint of the DNA fragment containing the adjacent genes. In surface motility assays, the *P. stewartii* wild-type strain showed either uni-directional (Fig. 3A and Fig. S1A) or omni-directional (Fig. 3B and Fig. S1B) expansion from the inoculum sites as had been previously observed (Herrera et al., 2008; Kernell Burke et al., 2015). In comparison to the wild type, there is no obvious difference between the various deletion and complementation strains; they all possessed similar level of expansion on the agar surface (Figs. S1C –S1J). Therefore, these genes do not appear to play any detectable role in surface motility via this assay.

The same deletion and complementation strains of the genes putatively involved in fimbriae and surfactant production were also tested for virulence via *in planta* xylem infection assays. A *lrhA* deletion strain caused an intermediate level of disease severity in corn seedlings during xylem-infection assays (Kernell Burke et al., 2015). However, similar to the surface motility assays, no significant impacts on the virulence of *P. stewartii* were observed in the strains with deletions in either the fimbriae or surfactant synthesis genes (Fig. S2). Hence, the contribution of these genes individually to the virulence of the phytopathogen could not be measured.

### Re-examining the role of RcsA in the capsule production, surface motility and virulence of *P. stewartii*

The important finding that LrhA directly binds to the promoter of *rcsA*, led to a reexamination of the previous findings about the physiological role of RcsA in *P. stewartii*. In prior work, *rcsA* deletion and complementation strains of DC283 had been constructed (Δ*rcsA*-2015 and Δ*rcsA/rcsA*^+^-2015) (Kernell Burke et al., 2015). However, complete assembly of the genome of *P. stewartii* DC283 (Duong, Stevens & Jensen, 2017) revealed that there is a large deletion, ∼66 kb containing 68 genes (Table S2), in the Δ*rcsA*-2015 and Δ*rcsA/rcsA*^+^-2015 strains. This deletion was not obvious using the incomplete genome sequence (NCBI GenBank accession no. AHIE00000000.1), but was found during a re-analysis of previously generated RNA-Seq data (Kernell Burke et al., 2015) using the new genome sequence (NCBI GenBank accession no. CP017581).

**Figure 4:**
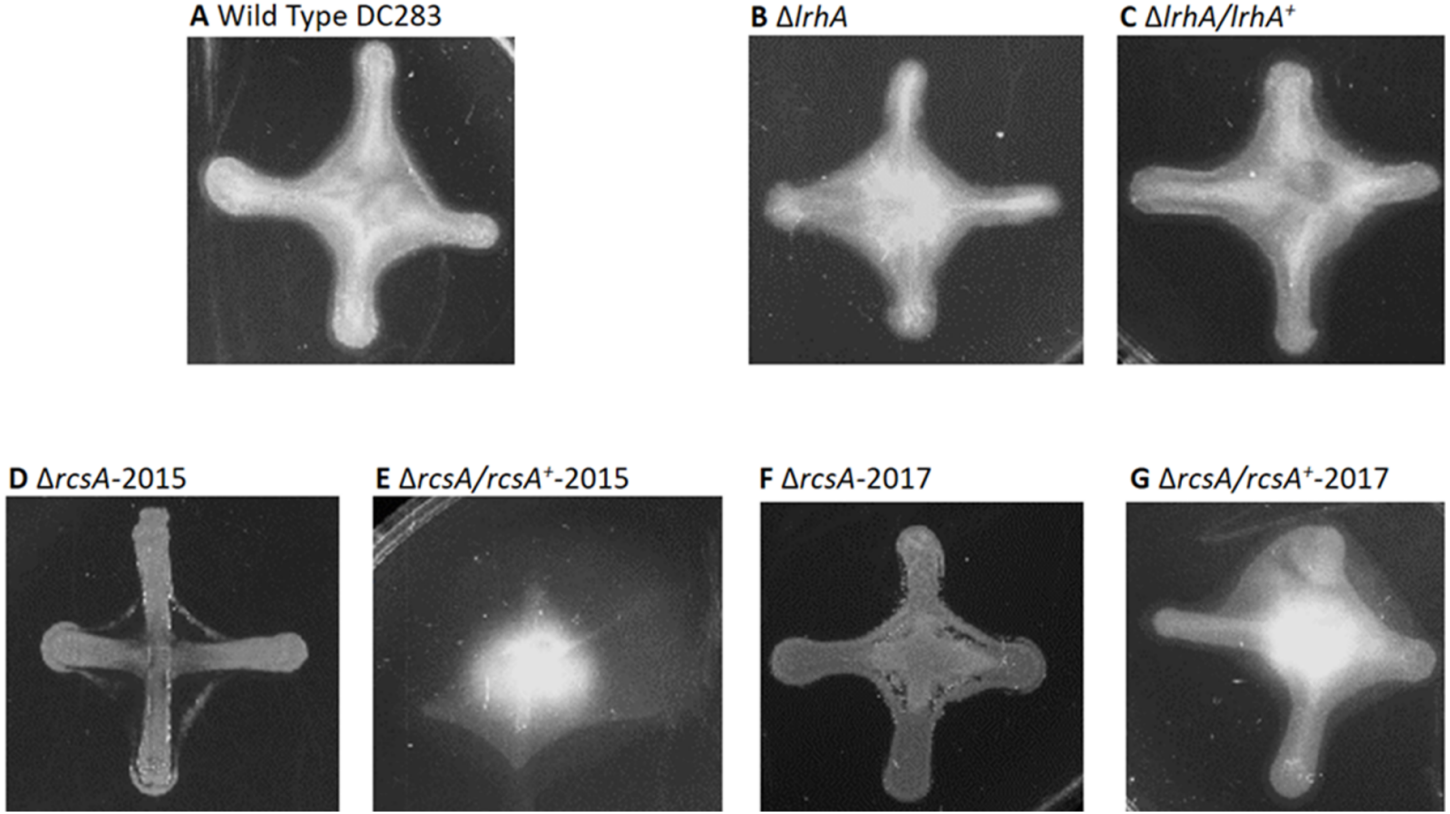
Impact of RcsA and LrhA on capsule production of *P. stewartii*. All pictures were taken at the same magnification after two days of incubation at 30 °C after cross-streaking on casamino acid, peptone, glucose (CPG) agar plates. Differences in capsule production are apparent in the regions between the arms of the X-cross streak.

Therefore, a new set of *rcsA* deletion and complementation strains was re-constructed (Δ*rcsA*-2017 and Δ*rcsA/rcsA*^+^-2017) and shown to include the 66-kb region using PCR (data not shown). These new strains then were subjected to three assays to establish the true phenotypes of the *rcsA* deletion strain. First, capsule production assays have re-confirmed that RcsA regulates EPS production, as was shown for the 2015 strains (Kernell Burke et al., 2015). Both the Δ*rcsA*-2015 (Fig. 4D) and Δ*rcsA*-2017 strains (Fig. 4F) are not as mucoid as the parental wild-type strain (Fig. 4A) or the pair of *lrhA* deletion and complementation strains (Figs. 4B and 4C) as assessed by visual observation. The chromosomal complementation strains Δ*rcsA/rcsA*^+^-2015 (Fig. 4E) and Δ*rcsA/rcsA*^+^-2017 (Fig. 4G) had mucoid levels as high or higher than those seen in the wild type (Fig. 4A).

Second, surface motility assays were performed. These original strains Δ*rcsA*-2015 and Δ*rcsA/rcsA*^+^-2015 strains had not previously been examined for surface motility (Kernell Burke et al., 2015), but both were surprisingly defective for this phenotype (Figs. 3E and 3F). The fact that surface motility was not complemented by addition of *rcsA* back into the chromosome provided further evidence for the importance of the 66-kb region deletion that had been discovered initially through bioinformatics analysis. The new Δ*rcsA*-2017 also has severely reduced surface movement (Fig. 3G) while its complementation strain (Fig. 3H) restored motility levels similar to the wild-type strain (Figs. 3A and 3B). Thus, it has been demonstrated that RcsA plays a previously unappreciated role in the surface motility of *P. stewartii.* The defect in surface motility associated with the deletion of *rcsA* (Fig. 3G) appears to be greater than the defect in Δ*lrhA* (Fig. 3C). The Δ*lrhA/lrhA*^+^ strain has restored levels of surface motility (Fig. 3D), similar to the wild type (Figs. 3A and 3B), as previously reported (Kernell Burke et al., 2015).

**Figure 5:**
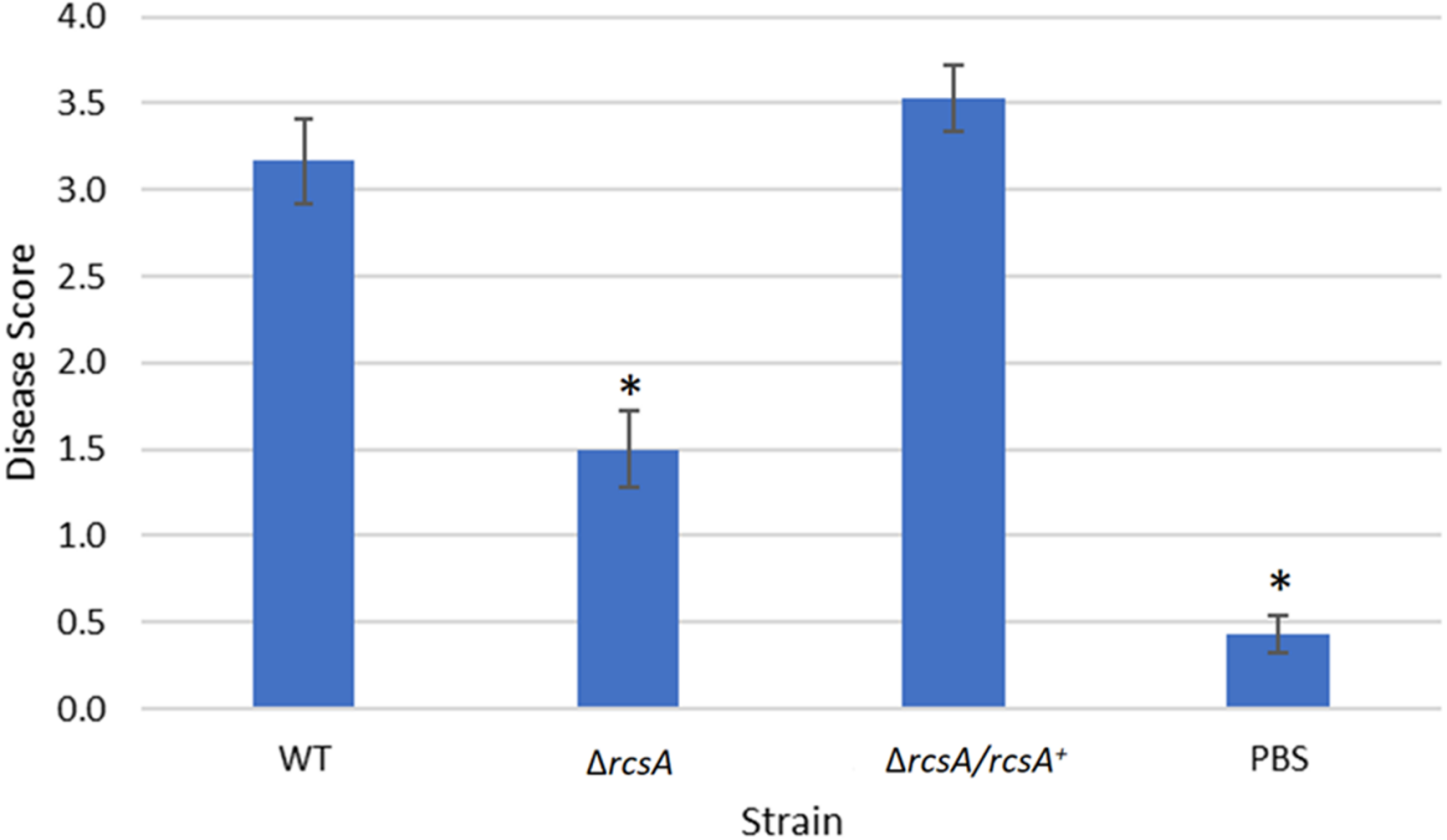
Plant assay testing the role of RcsA in virulence. Data shown is the average score of disease for Day 12 of an infection assay performed with 15 plants inoculated with *P. stewartii* DC283 strains: wild type (WT), Δ*rcsA*-2017, Δ*rcsA/rcsA*^+^-2017*,* or PBS as a negative control. Higher value in the disease score indicates more severe symptoms from the infection. The asterisks (∗) represent strains that are statistically significantly different (*p* < 0.05) from the wild-type strain using a two-tailed homoscedastic Student’s *t*-test. Error bars denote standard error.

Finally, the xylem infection assays for the newly constructed Δ*rcsA*-2017 strain and its complement, with the inclusion of the wild-type strain and PBS as controls, indicated that the absence of *rcsA* significantly (*p* < 0.05) reduces the severity of the disease compared to the wild-type and complementation strains (Fig. 5) (*p* < 0.05). These results have similar trends with those reported for the 2015 strains (Kernell Burke et al., 2015) which confirms the role of *rcsA* in virulence of this phytopathogen. However, strains from the 2015 study that were missing the 66-kb region were reduced in their average disease severity (score ∼0 and ∼1.5 for the deletion and complementation strains, respectively) in comparison to the new 2017 strains (score ∼1.5 and ∼3.5 for the deletion and complementation strains, respectively) while the wild-type control had similar levels in both studies, implicating a role for the 66-kb region in virulence as well as surface motility.

## Discussion

The role of the LrhA regulon in *P. stewartii* was further investigated in this study to understand how it is involved in the surface motility and virulence of the pathogen. Previous studies showed that surface motility in *P. stewartii* contributes to disease pathogenesis and this process involves both QS-controlled biofilm formation and flagella (Herrera et al., 2008). However, to date, there is no clear evidence to directly connect the synthesis of flagella to QS control in *P. stewartii*. Unlike *E. coli*, the QS-controlled transcription factor LrhA in *P. stewartii* does not regulate FlhD_2_C_2_, the master activator of flagellar synthesis. This was suggested by earlier RNA-Seq data (Kernell Burke et al., 2015), but directly tested here through EMSA that confirmed the inability of LrhA to bind to the *flhD/flhC* promoter. Additionally, LrhA activates its own expression in *E. coli* whereas autorepression was observed in *P. stewartii*. Even though *P. stewartii* LrhA has 77% amino acid identity to *E. coli* LrhA, the two have clearly evolved distinctive physiological roles in their host organisms.

In an attempt to define the function of the genes controlled by LrhA in *P. stewartii,* a reverse genetics approach was used to examine the role of select LrhA-regulated genes in surface motility and virulence of the phytopathogen. Multiple deletion and complementation strains of genes annotated as being involved in surfactant production (*CKS_5208* and *CKS_5211*, initially annotated as a rhamnosyltransferase I subunit B and putative alpha/beta superfamily hydrolase/acyltransferase, respectively) and fimbriae assembly (*CKS_0458* and *CKS_0459*, annotated as putative fimbrial subunits) were constructed and tested. Interestingly, none of these genes appear to play a fundamental role in surface motility and virulence individually. A LrhA deletion mutant impacting expression of multiple genes in the regulon produced noticeably decreased surface motility, but only intermediate virulence levels in comparison to the wild-type strain (Kernell Burke et al., 2015).

**Figure 6:**
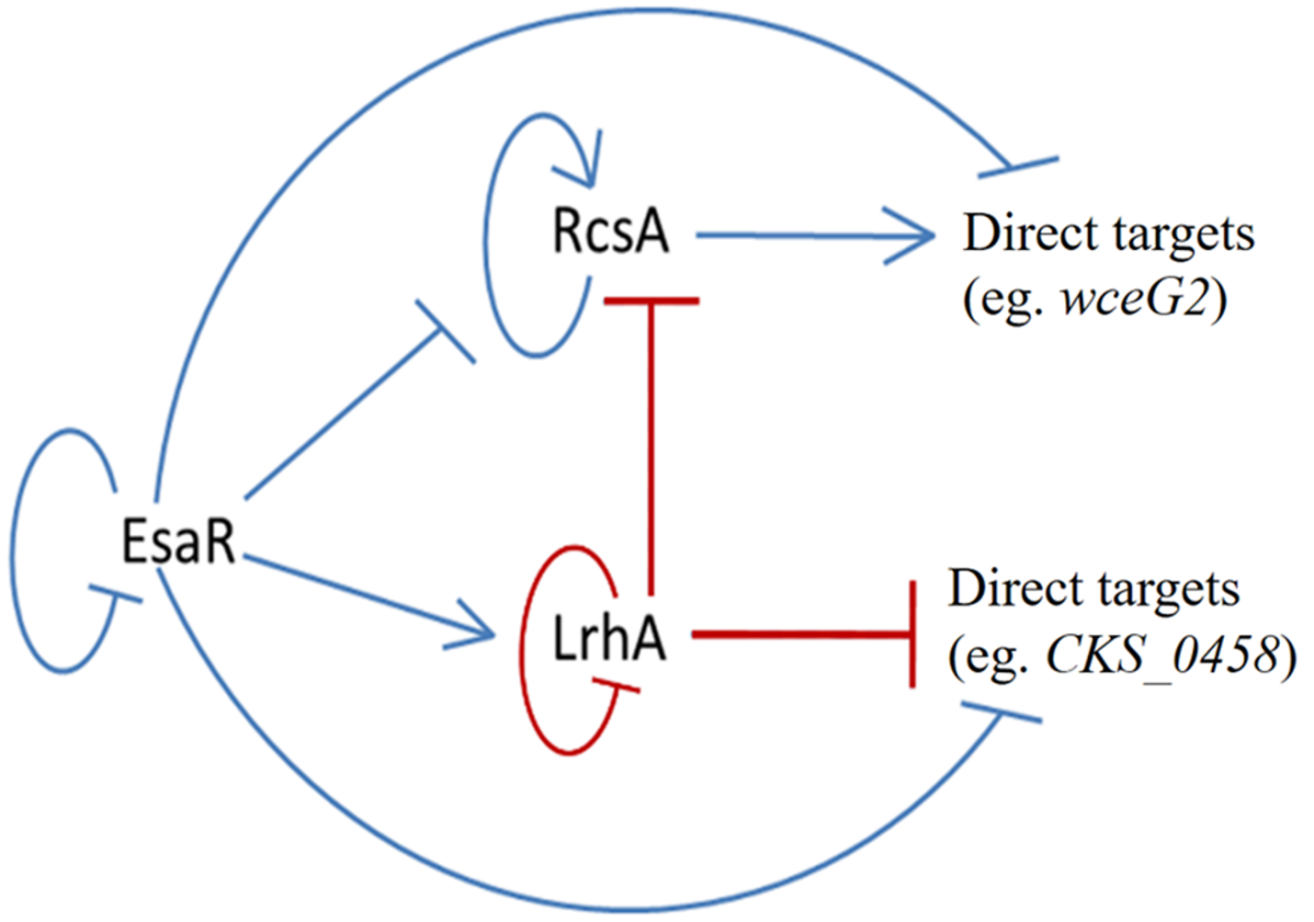
Updated model of the quorum-sensing regulatory network in *P. stewartii*. Solid lines indicate known direct regulatory control. Red lines indicate direct control found in this study. Arrows represent activation and *T* lines represent repression. At low cell density when AHL levels are low, EsaR represses expression of *rcsA, wceG2,* and *CKS_0458*,** and activates expression of *lrhA.* LrhA represses its own expression as well as that of *rcsA* and *CKS_0458.* At high cell density when EsaR-AHL complexes form, EsaR no longer activates or represses its direct targets. Thus, *rcsA* expression increases leading to activation of *wceG2* and other genes necessary for capsule production. See the text for additional details.

With regard to biosurfactant and fimbriae genes potentially associated with surface motility and adhesion, respectively, *P. stewartii* appears to utilize multiple levels of repression to ensure that the level of those genes’ expression is minimal. This low level of expression was again confirmed by an *in planta* RNA-Seq analysis (Packard et al., 2017). In the LrhA deletion strain expression of these genes was elevated. Thus, deletion mutants might actually mimic wild-type levels of the expression of these genes, producing a wild-type phenotype. Alternatively, these genes are not functional in the wild-type strain (indeed the new genome sequence (Duong, Stevens & Jensen, 2017) suggests that *CKS_5211* is a pseudogene) or they may serve another function for the bacterium that was not examined in this study. Biofilm/adhesion assays were inconclusive (data not shown). Interestingly, some *Pantoea* species have been demonstrated to produce biosurfactants when grown on hydrocarbons (Vasileva-Tonkova & Gesheva, 2007). How this might impact bacterial surface motility or survival *in planta* is unclear.

It has been demonstrated that both RcsA and LrhA play an essential role to the surface motility of the wild-type strain of *P. stewartii*. The observed intermediate impact of a LrhA deletion on virulence may be due primarily to its direct control of RcsA and thereby its indirect control on the levels of stewartan extracellular polysaccharide produced during growth within the plant. However, it could be that some of the other genes regulated by LrhA that were not examined in this work were actually contributing to the observed phenotypes in the LrhA deletion strain. RNA-Seq analysis of the transcriptome controlled by LrhA revealed 23 additional genes, in addition to the ones examined in this study, that were differentially expressed four-fold or more in comparison to the wild-type strain (Kernell Burke et al., 2015). Overall, the majority of the genes in the LrhA regulon code for hypothetical proteins and phage-related proteins, 57.7% (15/26) and 15.4% (4/26) respectively. The possible role of these genes with regard to surface motility and virulence remains to be established, but LrhA clearly regulates these processes.

The newly discovered connection between RcsA and surface motility suggests coordination of the RcsA and LrhA regulons with regard to bacterial virulence in the corn host beyond promotion of capsule production. Capsule production is thought to be a factor impacting the ability of surface motility to occur in this phytopathogen, which may explain the need for integrated downstream regulation. The fact that the strain with the 66-kb deletion region could not be complemented by *rcsA* suggests that there are additional genes in this region that are essential to surface motility and virulence. Further work will be needed to identify these genes and to overall correlate to the ability of the phytopathogen to move inside the plant via surface motility in relation to virulence.

## Conclusions

The findings of this study have further defined the tightly coordinated gene regulation that occurs in the QS regulon of the corn pathogen *P. stewartii.* The EsaR-activated transcription factor LrhA was found to directly auto-repress expression of its own gene as demonstrated through GFP-transcription fusions and EMSA experiments. In addition, the direct binding of LrhA to downstream targets, such as the promoters of genes coding for RcsA*,* and for putative biosurfactant synthesis (*CKS_5211*) and fimbrial production (*CKS_0458*), was also shown. This established a hierarchy of gene regulation in the QS network from the master regulator, EsaR, to the downstream transcription factors, RcsA and LrhA, which in turn control the expression of their own targets. Intriguingly, EsaR also directly controls some of these same targets (Ramachandran et al., 2014; Ramachandran & Stevens, 2013) integrating with coherent type two (RcsA) and type three (LrhA) feed forward loops (Mangan & Alon, 2003) to regulate genes in the QS regulon in a manner that ensures precisely synchronized gene expression (Fig. 6).

##  Supplemental Information

10.7717/peerj.4145/supp-1 Table S1 Primers used for strain construction

10.7717/peerj.4145/supp-2 Table S2 List of 68 genes present in the 66-kb deletion region in Δ*rcsA*-2015

10.7717/peerj.4145/supp-3 Figure S1 Impact of putative fimbrial and surfactant genes on surface motility Surface motility assays for the indicated strains. All pictures were taken at the same magnification after 48 hours of incubation.

10.7717/peerj.4145/supp-4 Figure S2 Xylem-infection assays testing the role of putative fimbrial and surfactant genes in virulence Data shown is the average score of disease for Day 12 of an infection assay performed with 15 plants inoculated with *P. stewartii* DC283 strains or PBS as a negative control as indicated on the X-axis. Error bars denote standard errors. The asterisk (∗) indicates a statistically significant difference (*p* < 0.05) between the wild type and the negative control while the remaining strains have *p* > 0.05 using a two-tailed homoscedastic Student’s t-test.

10.7717/peerj.4145/supp-5 Data S1 Data file for bar graphs

## Additional Information and Declarations

### Data Availability

The following information was supplied regarding data availability: The raw data has been provided as Data S1.
