# Supplementary material for: Integrated downstream regulation by the quorum-sensing controlled transcription factors LrhA and RcsA impacts phenotypic outputs associated with virulence in the phytopathogen Pantoea stewartii subsp. stewartii
Source: PeerJ. 2017 Dec 6;5:e4145. doi: 10.7717/peerj.4145 (PMC5723134; doi:10.7717/peerj.4145)
Supplement: Table S1 [file peerj-05-4145-s001.docx]

**Table S1. Primers used for strain construction.**

| **Primers** | **Sequence (5’ to 3’)** | **Purpose** |
| --- | --- | --- |
| **GFP transcriptional fusion** | | |
| EcoRI-PlrhA-F | GAATTCTGCACAATGTACTCTCCTCACG | GFP transcriptional fusion construction |
| KpnI- PlrhA-R | GGTACCGAGTTTTAAGAACTACTATCCTGG |  |
| **LrhA overexpression** | | |
| BamHI-LrhA-F | GGATCCATGACTAATGCAAATCGTCCG | *lrhA* coding sequence cloning for overexpression |
| HindIII-LrhA-R | AAGCTTCTATTACTCTTCATCGTCCAGCAG |  |
| **Targets of LrhA for EMSA** | | |
| FAM_PlrhA_R | /FAM/GAGTTTTAAGAACTACTATCCTGG | Generation of P*_lrhA_*-FAM probe |
| SpeI-LrhA-F | ACTAGTCATAGCGTAAGTAGGGTGTGAC |  |
| FAM_PrcsA_R | /FAM/GTTAGCGACCCTCACCAATTTGTTATCC | Generation of P*_rcsA_*-FAM probe |
| TFRcsAFwd  EcoRI (Kernell Burke et al. 2015) | GAATTGGAAATTCACAACTATCCGGGCATTTTTC |  |
| PflhDC-R-FAM | /FAM/ATCCTGAGAGACGCGACGTAACGG | Generation of P*_flhDC_*-FAM probe |
| PflhDC-F2 | GACCGGACAGACAGGGTCGC |  |
| P5211-R-FAM | /FAM/CGTGACTCATATCACTCTCCCCACC | Generation of P*_CKS_5211_*-FAM probe |
| P5211-F1 | CGGTGTTTCACGCGATAGTGTCC |  |
| P5208-R-FAM | /FAM/CGCCAATAATCACTTTCATCTCAGACACC | Generation of P*_CKS_5208_*-FAM probe |
| P5208-F | GCTGTGGTAGGCTTTGTTGAATAAATGG |  |
| P0458-R-FAM | /FAM/TAGCAACACCGGCAGCTAACAG | Generation of P*_CKS_0458_*-FAM probe |
| P0458-F | GATTTCCGAATAGCGCCGAACACG |  |
| BamHI- PlrhA-R | GGATCCGAGTTTTAAGAACTACTATCCTGG | Generation of P*_lrhA_* competitor |
| SpeI-LrhA-F | ACTAGTCATAGCGTAAGTAGGGTGTGAC |  |
| **Deletion construction** | | |
| CKS_0458/CKS_0459-UPF | GTCGACATCGCGACAATGCCGCAGC | Amplify 1 kb region upstream of *CKS_0458/CKS_0459* |
| CKS_0458/CKS_0459-UPR | AGTGGAATATAGGCGGCCGCAAGCTTGAATTTCATAACC |  |
| CKS_0458/CKS_0459-DNF | GCGGCCGCCTATATTCCACTGTCACGCTGACCTGGTGA | Amplify 1 kb region downstream of *CKS_0458/CKS_0459* |
| CKS_0458/CKS_0459-DNR | GGATCCCGTTGTAAGTCGCCTGCCCGC |  |
| CKS_0458/CKS_0459-1kbUPF-attB1 | GGGGACAAGTTTGTACAAAAAAGCAGGCTGTCGACATCGCGACAATGCCGCAGC | Amplify 2 kb deletion fragment of *CKS_0458/CKS_0459* with flanking *attB* sites |
| CKS_0458/CKS_0459-1kbDNR-attB2 | GGGGACCACTTTGTACAAGAAAGCTGGGTGGATCCCGTTGTAAGTCGCCTGCC |  |
| UP-CKS_0458/CKS_0459-SeqF | GGCGCTGGAGAGAGTGACGG | Screen/sequence mutants for *CKS_0458/CKS_0459* deletion |
| IN-CKS_0458/CKS_0459-SeqF | GTGCCTGTGTTGGCGTAGATGC |  |
| DN-CKS_0458/CKS_0459-SeqR | CCCTGAGCCTGAGGCAACACC |  |
| CKS_5208-UPF | GTCGACGAGAGTGATATGAGTCACGG | Amplify 1 kb region upstream of *CKS_5208* |
| CKS_5208-UPR | AGTGGAATATAGGCGGCCGCCACTTTCATCTCAGACACC |  |
| CKS_5208-DNF | GCGGCCGCCTATATTCCACTTAAACGCGTCTTCAGGC | Amplify 1 kb region downstream of *CKS_5208* |
| CKS_5208-DNR | GGATCCGATTTCCCGCAGTATTTCTCG |  |
| CKS_5208-1kbUPF-attB1 | GGGGACAAGTTTGTACAAAAAAGCAGGCTGTCGACGAGAGTGATATGAGTCACGG | Amplify 2 kb deletion fragment of *CKS_5208* with flanking *attB* sites |
| CKS_5208-1kbDNR-attB2 | GGGGACCACTTTGTACAAGAAAGCTGGGTGGATCCGATTTCCCGCAGTATTTCTCG |  |
| UP-CKS_5208-SeqF | ATCTTGAGCAGATTGCCACGC | Screen/sequence of mutants for *CKS_5208* deletion |
| IN-CKS_5208-SeqF | CTGTGCAACTGGCTAATCAAACCC |  |
| DN-CKS_5208-SeqR | GCGTCACTGGCACAGTATATGG |  |
| CKS_5211-UPF | GTCGACCCAGTTGAACAGGAGATTATCG | Amplify 1 kb region upstream of *CKS_5211* |
| CKS_5211-UPR | AGTGGAATATAGGCGGCCGCGTTTACTACAGAATAACCGTG |  |
| CKS_5211-DNF | GCGGCCGCCTATATTCCACTGATTTTATGCTGTGGTAGGC | Amplify 1 kb region downstream of *CKS_5211* |
| CKS_5211-DNR | GGATCCGGGTTCGTATAACACAATCG |  |
| CKS_5211-1kbUPF-attB1 | GGGGACAAGTTTGTACAAAAAAGCAGGCTGTCGACCCAGTTGAACAGGAGATTATCG | Amplify 2 kb deletion fragment of *CKS_5211* with flanking *attB* sites |
| CKS_5211-1kbDNR-attB2 | GGGGACCACTTTGTACAAGAAAGCTGGGTGGATCCGGGTTCGTATAACACAATCG |  |
| UP-CKS_5211-SeqF | ATAAGACCAGCCTCCCTTTCCTCG | Screen/sequence of mutants for *CKS_5211* deletion |
| IN-CKS_5211-SeqF | GTGTCCCGACCCGTAACAGG |  |
| DN-CKS_5211-SeqR | CCGTCATACGCAAGCATGTAACGC |  |
| **Chromosomal complementation construction** | | |
| SacI-CKS_0458/CKS_0459-F | GAGCTCAGTGATTTCCGAATAGCGCCG | Amplify promoter and coding region of *CKS_0458/CKS_0459,* and screen conjugants |
| XhoI- CKS_0458/CKS_0459-R | CTCGAGCTATCACCAGGTCAGCGTGAC |  |
| XhoI- CKS_0458-R | CTCGAGCTACACTTAATAGTTCACGGCAACG | Work with SacI-CKS_0458/CKS_0459-F to amplify promoter and coding region of *CKS_0458,* and screen conjugants |
| SacI-CKS_5208-F | GAGCTCAACGGCGACCTGGATATGGC | Amplify promoter and coding region of *CKS_5208,* and screen conjugants |
| XhoI-CKS_5208-R | CTCGAGCTATTAACGGGTCAGAAAGCGTTCC |  |
| SacI-CKS_5211-F | GAGCTCATCGACTGCTCCACCATGG | Amplify promoter and coding region of *CKS_5211,* and screen conjugants |
| XhoI-CKS_5211-R | CTCGAGCTATCAACAAAGCCTACCACAGC |  |
