# Supplementary material for: Integrated downstream regulation by the quorum-sensing controlled transcription factors LrhA and RcsA impacts phenotypic outputs associated with virulence in the phytopathogen Pantoea stewartii subsp. stewartii
Source: PeerJ. 2017 Dec 6;5:e4145. doi: 10.7717/peerj.4145 (PMC5723134; doi:10.7717/peerj.4145)
Supplement: Table S2 [file peerj-05-4145-s002.docx]

**Table S2. List of 68 genes present in the 66-kb deletion region in Δ*rcsA*-2015.**

| **locus_tag** | **Annotated Name** | **Length (bp)** | **Coding**  **Strand** |
| --- | --- | --- | --- |
| DSJ_09100 | IS630 family transposase | 1054 | forward |
| DSJ_09105 | histidine utilization repressor | 756 | forward |
| DSJ_09110 | HutD family protein | 546 | reverse |
| DSJ_09115 | formimidoylglutamate deiminase | 1356 | reverse |
| DSJ_09120 | imidazolonepropionase | 1218 | forward |
| DSJ_09125 | N-formylglutamate deformylase | 789 | forward |
| DSJ_09130 | Asp/Glu/hydantoin racemase | 636 | reverse |
| DSJ_09135 | NAD(P)H-binding protein | 1107 | forward |
| DSJ_09140 | 23S rRNA (adenine(1618)-N(6))-methyltransferase | 924 | forward |
| DSJ_09145 | glnQ | 723 | reverse |
| DSJ_09150 | glutamine ABC transporter permease GlnP | 660 | reverse |
| DSJ_09155 | glutamine ABC transporter substrate-binding protein GlnH | 747 | reverse |
| DSJ_09160 | DNA starvation/stationary phase protection protein Dps | 504 | reverse |
| DSJ_09165 | threonine/homoserine exporter RhtA | 912 | reverse |
| DSJ_09170 | ompX | 513 | forward |
| DSJ_09175 | hypothetical protein | 186 | reverse |
| DSJ_09180 | transcriptional regulator MntR | 459 | forward |
| DSJ_09185 | anion transporter | 1110 | forward |
| DSJ_09190 | multidrug export protein EmrA | 1149 | forward |
| DSJ_09195 | EmrB/QacA family drug resistance transporter | 1569 | forward |
| DSJ_09200 | RND transporter | 1530 | forward |
| DSJ_09205 | DNA-binding protein | 402 | forward |
| DSJ_09210 | ABC-F family ATPase | 1593 | forward |
| DSJ_09215 | molybdopterin-synthase adenylyltransferase MoeB | 768 | reverse |
| DSJ_09220 | molybdopterin molybdotransferase | 1236 | reverse |
| DSJ_09225 | beta-aspartyl-peptidase | 960 | forward |
| DSJ_09230 | glutathione ABC transporter ATP-binding protein GsiA | 1830 | forward |
| DSJ_09235 | glutathione ABC transporter substrate-binding protein GsiB | 1536 | forward |
| DSJ_09240 | glutathione ABC transporter permease GsiC | 927 | forward |
| DSJ_09245 | glutathione ABC transporter permease GsiD | 906 | forward |
| DSJ_09250 | cytoplasmic protein | 450 | forward |
| DSJ_09255 | rimO | 1329 | reverse |
| DSJ_09260 | carbonic anhydrase | 636 | forward |
| DSJ_09265 | oxidoreductase | 1137 | forward |
| DSJ_09270 | hydrolase | 657 | reverse |
| DSJ_09275 | serine-type D-Ala-D-Ala carboxypeptidase | 1203 | forward |
| DSJ_09280 | undecaprenyl-diphosphate phosphatase | 606 | reverse |
| DSJ_09285 | transporter | 1689 | reverse |
| DSJ_09290 | hypothetical protein | 366 | forward |
| DSJ_09295 | glutaredoxin | 264 | reverse |
| DSJ_09300 | hypothetical protein | 255 | forward |
| DSJ_09305 | nitroreductase A | 723 | forward |
| DSJ_09310 | ribosomal protein S6 modification protein | 903 | forward |
| DSJ_09315 | sensory transduction regulator | 480 | forward |
| DSJ_09320 | IS66 family transposase | 1572 | reverse |
| DSJ_09325 | transposase | 348 | reverse |
| DSJ_09330 | transposase | 630 | reverse |
| DSJ_09335 | spermidine/putrescine ABC transporter substrate-binding protein PotF | 1110 | forward |
| DSJ_09340 | potG | 1134 | forward |
| DSJ_09345 | putrescine ABC transporter permease PotH | 963 | forward |
| DSJ_09350 | putrescine ABC transporter permease PotI | 846 | forward |
| DSJ_09355 | hypothetical protein | 468 | forward |
| DSJ_09360 | rumB | 1146 | forward |
| DSJ_09365 | arginine ABC transporter substrate-binding protein | 732 | reverse |
| DSJ_09370 | artM | 669 | reverse |
| DSJ_09375 | arginine transporter permease subunit ArtQ | 717 | reverse |
| DSJ_09380 | arginine ABC transporter substrate-binding protein | 732 | reverse |
| DSJ_09385 | arginine ABC transporter ATP-binding protein ArtP | 750 | reverse |
| DSJ_09390 | lipoprotein | 540 | reverse |
| DSJ_09395 | hypothetical protein | 321 | forward |
| DSJ_09400 | N-acetylmuramoyl-L-alanine amidase | 825 | forward |
| DSJ_09405 | low-specificity L-threonine aldolase | 1062 | reverse |
| DSJ_09410 | pyruvate oxidase | 1722 | reverse |
| DSJ_09415 | hypothetical protein | 900 | reverse |
| DSJ_09420 | hypothetical protein | 306 | forward |
| DSJ_09425 | ATP-dependent endonuclease | 1344 | forward |
