# Supplementary figures and images for: Integrated downstream regulation by the quorum-sensing controlled transcription factors LrhA and RcsA impacts phenotypic outputs associated with virulence in the phytopathogen Pantoea stewartii subsp. stewartii
Source: PeerJ. 2017 Dec 6;5:e4145. doi: 10.7717/peerj.4145 (PMC5723134; doi:10.7717/peerj.4145)

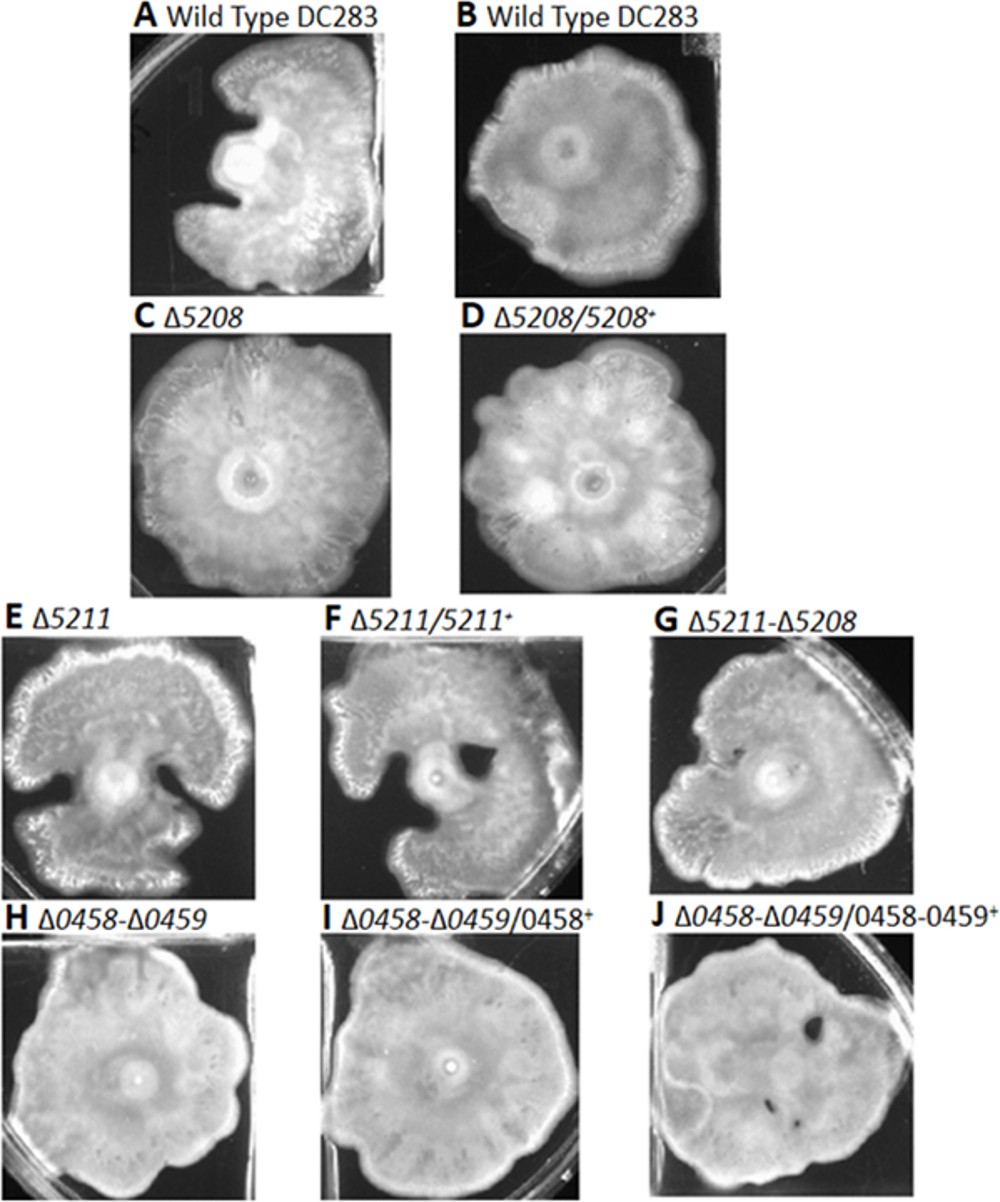

Supplement: Figure S1 — Surface motility assays for the indicated strains. All pictures were taken at the same magnification after 48 hours of incubation. [file peerj-05-4145-s003.png]

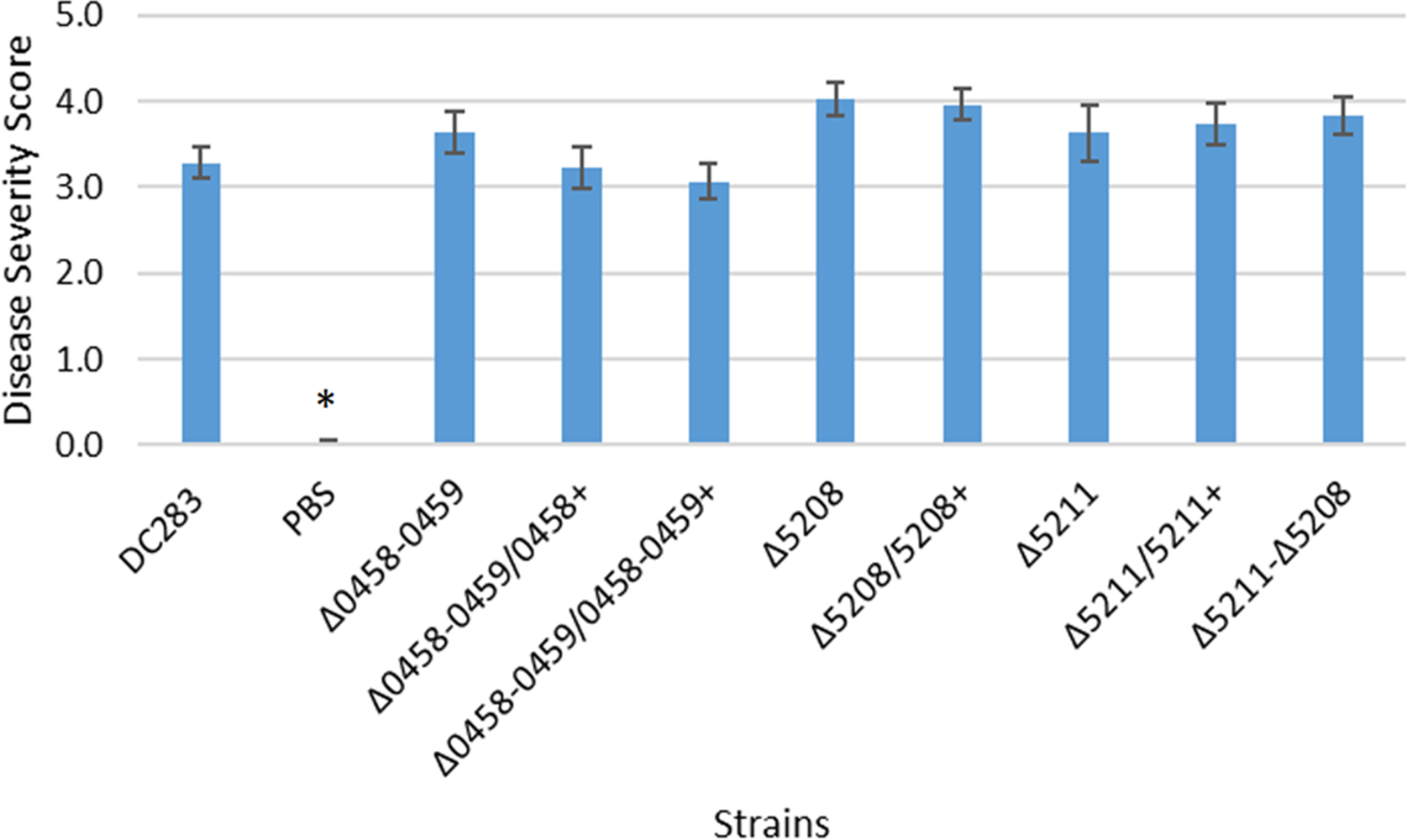

Supplement: Figure S2 — Data shown is the average score of disease for Day 12 of an infection assay performed with 15 plants inoculated with P. stewartii DC283 strains or PBS as a negative control as indicated on the X-axis. Error bars denote standard errors. The asterisk (∗) indicates a statistically significant difference (p < 0.05) between the wild type and the negative control while the remaining strains have p > 0.05 using a two-tailed homoscedastic Student’s t-test. [file peerj-05-4145-s004.png]
